# Association between high-density lipoprotein cholesterol level and pulmonary function in healthy Korean adolescents: the JS high school study

**DOI:** 10.1186/s12890-017-0548-6

**Published:** 2017-12-11

**Authors:** Ji Hye Park, Seyeon Mun, Dong Phil Choi, Joo Young Lee, Hyeon Chang Kim

**Affiliations:** 1 Department of Public Health, Yonsei University Graduate School, Seoul, South Korea; 2 Cardiovascular and Metabolic Diseases Etiology Research Center, Yonsei University College of Medicine, Seoul, South Korea; 3 Department of Preventive Medicine, Yonsei University College of Medicine, 50-1 Yonsei-ro, Seodaemun-gu, Seoul, 120-752 Republic of Korea

**Keywords:** High-density lipoprotein, Respiratory function tests, Forced vital capacity, Forced expiratory volume, Adolescent

## Abstract

**Background:**

Accumulating evidence suggests that high-density lipoprotein (HDL) cholesterol is associated with pulmonary function and pulmonary disorders. The aim of this study was to evaluate the association between HDL cholesterol and pulmonary function in healthy adolescents.

**Methods:**

This cross-sectional study was based on data collected for the JS High School study. The analysis included 644 adolescents (318 male and 326 female) aged 15–16 years old and free from asthma or chronic obstructive pulmonary disease. Fasting blood samples were collected for hematologic and biochemical assessment. Forced vital capacity volume (FVC) and forced expiratory volume in the 1 s (FEV1) were measured using dry-rolling-seal spirometry. The associations between HDL cholesterol and pulmonary function were analyzed using multiple linear regression models.

**Results:**

Among male adolescents, an increase of 1.0 mg/dL in HDL cholesterol was associated with 10 mL decrease in FVC (*p* = 0.013) and FEV1 (p = 0.013) after adjusting for age, height, weight, alcohol drinking, smoking, physical activity, systolic blood pressure, total cholesterol, triglyceride, and monthly household income. Percent predicted values of FVC (*p* = 0.036) and FEV1 (*p* = 0.017) were also inversely associated with HDL cholesterol. However, among female adolescents, HDL cholesterol level was not significantly associated with absolute or percent predictive value of FVC and FEV1.

**Conclusions:**

Higher HDL cholesterol level may be associated with decreased pulmonary function among healthy male adolescents. The sex differences observed in the association between HDL cholesterol and pulmonary function need further investigation.

**Electronic supplementary material:**

The online version of this article (10.1186/s12890-017-0548-6) contains supplementary material, which is available to authorized users.

## Background

High-density lipoprotein (HDL) is one of the five major groups of lipoproteins. HDL is found associated with apolipoprotein M more than other lipoproteins for the reason that apolipoprotein M is important for the formation of pre-beta HDL and for reverse cholesterol transport [1, 2]. HDL cholesterol is known to reduce macrophage accumulation [3] and to transport fat molecules out of artery walls [4], thereby preventing atherosclerosis [5, 6] and cardiovascular disease [7, 8]. For this reason, HDL cholesterol as a therapeutic target for atherosclerotic disease seems attractive strategy towards preventing cardiovascular disease. However, recent clinical trials aimed at increasing HDL cholesterol levels failed to observe any prevention of cardiovascular disease [9, 10]. Similarly, in a Mendelian randomization study, genetically increased HDL cholesterol levels did not reduce the risk of myocardial infarction [11].

Recent studies suggest that HDL cholesterol and apolipoprotein M may be associated with pulmonary function [12, 13]. A pooled analysis of seven cohort studies indicated that higher HDL cholesterol levels were independently associated with poorer pulmonary function [12]. Higher HDL cholesterol level was also positively associated with per cent emphysema measured by CT scan in the Multi-Ethnic Study of Atherosclerosis [12]. Apolipoprotein M was elevated in patients with chronic obstructive pulmonary disease and the level increased gradually as disease severity increased [13]. These finding imply that HDL cholesterol plays a significant role in pulmonary function. However, to date no research has been conducted on the relationship between HDL cholesterol and pulmonary function in healthy adolescents. Therefore, in this study, we investigated the possible association between HDL cholesterol level and pulmonary function in healthy adolescents.

## Methods

### Study population

Our study was based on data collected for the JS High School study, a prospective cohort study of a Korean adolescent population [14]. The target population of the JS High School study was first-graders at a high school located in a rural area of South Korea. Baseline examinations were conducted on 1071 participants in years 2007, 2010, 2011, and 2012. Pulmonary function tests were conducted in years 2007, 2010, and 2011 and the reproducibility of the results was confirmed by a respiratory physician. Among the 830 participants who underwent pulmonary function testing, we selected 644 (318 male and 326 female) participants after excluding 170 participants who had poorly reproducible results and 16 participants who were diagnosed with and treated for asthma and respiratory diseases. Informed consent was obtained from each student as well as from his/her parent or guardian after full explanation of the purpose and process of the study. The study protocol and consent procedure were approved by the Institutional Review Board of Severance Hospital at Yonsei University College of Medicine.

### Measurements

Health-related lifestyle factors and personal and family disease history were evaluated using self-administered questionnaires (Additional file 1). Smokers were defined as participants who smoked more than 100 cigarettes in their lifetime. Drinkers were defined as participants who consumed alcoholic beverages at least once a month over the last year. Regular exercise was defined as engaging in physical activity on a regular basis, for at least 30 min once a week at moderate intensity, either indoor or outdoor. Monthly household income was classed into three groups: low (<3,000,000 Korean won per month), middle (3,000,000 to 5,000,000 Korean won per month), and high (>5,000,000 Korean won per month). Anthropometric measurements were performed according to a predefined protocol. Height was measured to the nearest 0.1 cm using a stadiometer. Body weight was measured to the nearest 0.1 kg on a digital scale, with the subject wearing his/her school uniform. Body mass index was calculated as weight in kilograms divided by the square of height in meters (kg/m^2^). Waist circumference was measured between the lower borders of the rib cage and the iliac crest with a measuring tape. Resting blood pressure was measured twice, at 5-min intervals, using an automatic sphygmomanometer (Dinamap 1846 SX/P, USA) with the participant in the sitting position. If the two readings differed by more than 10 mmHg, additional readings were obtained and the last two readings were averaged. Fasting blood samples were drawn after at least an 8-h fast. Serum concentrations of total cholesterol, HDL cholesterol, and triglycerides were measured by enzymatic methods using an autoanalyzer (ADVIA 1800, Siemens Healthcare Diagnostics Inc., Deerfield, IL, USA).

Pulmonary function testing was conducted using a volume displacement spirometer (model 1022; Sensor Medics; Yorba Linda, USA) after instruction and a practice attempt, in the seated position with nose clips applied. Participants performed at least three different forced expiration maneuvers to provide estimates of forced vital capacity (FVC) and forced expired volume in 1 s (FEV1), according to the guidelines of the American Thoracic Society and European Respiratory Society [15]. If the spirometry was judged as not being optimal or showing obstruction, the participants were asked to perform up to eight times. The best forced expiratory volume was recorded from each set of measurements. The percent predicted values of FVC (%FVC) and FEV1 (%FEV1) were calculated according to Polgar’s formula, correcting sex, age, height, and weight [16].

### Statistical analysis

Data are presented either as mean with standard deviation or number with percent. Differences between male and female participants were analyzed using either the independent *t*-test or χ^2^-test. Correlations between variables were evaluated using Spearman’s correlation coefficient. Multiple linear regression models were used to examine the association between HDL cholesterol level and pulmonary function, adjusting for potential confounding variables including age, height, weight, physical activity level, systolic blood pressure, and total cholesterol and triglyceride levels. All statistical analyses were performed using SAS version 9.2 (SAS Institute, Cary, NC, USA). Statistical significance was defined as a two-sided *p*-value less than 0.05.

## Results

The clinical and biochemical characteristics of participants are presented in Table 1. Pulmonary function indices such as FVC, %FVC, FEV1, and %FEV1 were higher in male participants. Total cholesterol, HDL cholesterol, and low-density lipoprotein (LDL) cholesterol were higher in female participants. The proportions of current smokers and drinkers were significantly higher among male participants, who also engaged more regularly in exercise than their female counterparts. No participants had been diagnosed with diabetes mellitus or hypertension.

**Table 1:** Participants’ characteristics

Variable	Males (*n* = 318)	Females (*n* = 326)	*p*-value
Mean ± standard deviation			
Age, year	15.8 ± 0.3	15.8 ± 0.3	0.997
Height, cm	171.2 ± 5.4	159.9 ± 5.0	<.001
Weight, kg	64.5 ± 10.3	54.1 ± 7.3	<.001
Body mass index, kg/m^2^	22.0 ± 3.1	21.1 ± 2.6	<.001
Waist circumference, cm	74.0 ± 8.1	69.2 ± 6.7	<.001
Systolic BP, mmHg	112.5 ± 11.6	102.8 ± 9.9	<.001
Diastolic BP, mmHg	59.3 ± 6.9	59.4 ± 6.7	0.954
Total cholesterol, mg/dL	148.4 ± 26.6	160.9 ± 25.8	<.001
HDL cholesterol, mg/dL	41.5 ± 8.6	47.5 ± 9.4	<.001
LDL cholesterol, mg/dL	90.3 ± 23.2	97.6 ± 22.6	<.001
Triglycerides, mg/dL	83.5 ± 31.3	78.9 ± 26.9	0.059
FVC, L	4.51 ± 0.62	3.23 ± 0.39	<.001
FEV1, L	3.98 ± 0.53	2.84 ± 0.40	<.001
Percent predicted FEV1	109.4 ± 11.5	90.6 ± 12.1	<.001
Percent predicted FVC	114.2 ± 12.8	98.3 ± 10.1	<.001
Number (%)			
Smoking (≥100 cigarettes)	15 (4.7)	3 (0.9)	0.004
Drinking (≥1 time/month)	34 (10.7)	16 (4.9)	0.006
Regular exercise (≥1 time/week)	281 (88.4)	259 (79.5)	0.002
Monthly household income			
< 3,000,000 won	61 (19.2)	51 (15.6)	0.622
3,000,000 to 5,000,000 won	101 (31.8)	112 (34.4)	
> 5,000,000 won	80 (25.2)	79 (24.2)	
Do not wish to answer	76 (23.9)	84 (25.8)	

Data presented as mean ± standard deviation or as n (%)

*Abbreviations*: *HDL* high density lipoprotein, *LDL* low density lipoprotein, *FVC* forced vital capacity, *FEV1* forced expiratory volume in one second

**Fig. 1:**
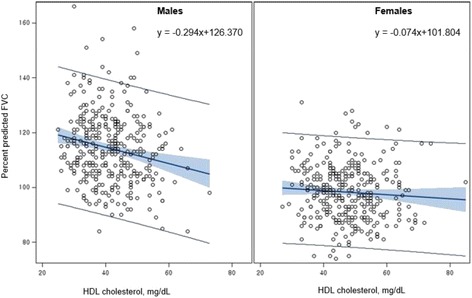
Association between HDL cholesterol level and percent predicted FVC

**Fig. 2:**
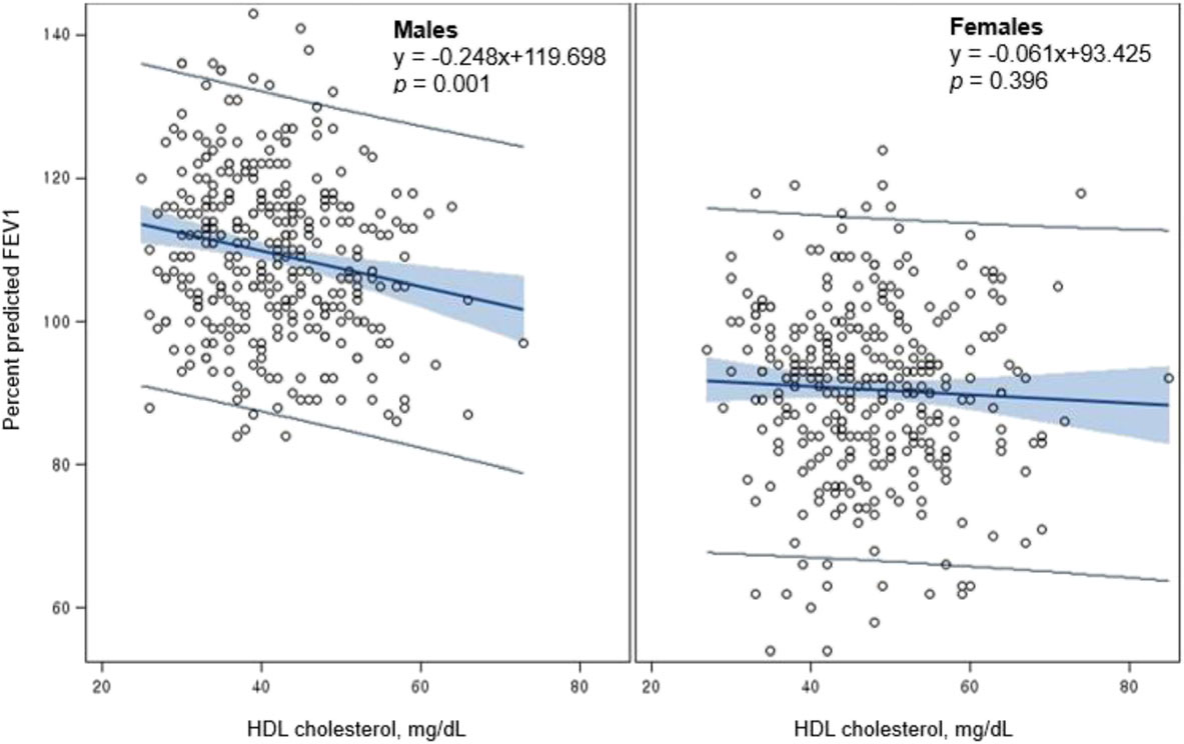
Association between HDL cholesterol level and percent predicted FEV1

Table 2 shows the correlations between HDL cholesterol level and pulmonary function indices, including FVC, FEV1, and FEV1/FVC ratio. HDL cholesterol showed significant negative correlations with values of FVC (*r* = −0.158, *p* = 0.005), FEV1 (*r* = −0.137, *p* = 0.015), %FVC (*r* = −0.172, *p* = 0.002), and %FEV1 (*r* = −0.165, *p* = 0.003) in male adolescents. However, in female adolescents, HDL cholesterol showed significant negative correlations with only %FVC (*r* = −0.112, *p* = 0.044). In both sexes, FEV1/FVC ratio was not correlated with HDL cholesterol level. Pulmonary function was positively correlated with BMI and systolic blood pressure in both male and female adolescents, but was only positively correlated with age in male participants. Figures 1 and 2 show the sex differences in the linear relationship between HDL cholesterol and %FVC and %FEV1, respectively. HDL cholesterol was significantly and inversely associated with %FVC and %FEV1 in male adolescents only. The scatterplots show a considerable sex difference in the association between HDL cholesterol and pulmonary function.

**Table 2:** Correlations between lung function and relevant factors, stratified by sex

Variable	FVC		FEV1		Percent predicted FVC		Percent predicted FEV1		FEV1/FVC, %	
	r	*p*	r	*p*	r	*p*	r	*p*	r	*p*
Men (*n* = 318)										
Age	0.135	0.016	0.149	0.008	0.193	0.001	0.208	<.001	0.027	0.637
Body mass index	0.338	<.001	0.203	<.001	0.455	<.001	0.293	<.001	−0.274	<.001
Systolic blood pressure	0.192	0.001	0.128	0.023	0.219	<.001	0.148	0.008	−0.116	0.039
Diastolic blood pressure	0.053	0.349	0.015	0.784	−0.013	0.812	−0.063	0.261	−0.058	0.305
Total cholesterol	−0.049	0.388	−0.044	0.431	−0.084	0.135	−0.070	0.216	−0.021	0.708
HDL cholesterol	−0.158	0.005	−0.137	0.015	−0.172	0.002	−0.165	0.003	0.037	0.514
Triglycerides	0.006	0.916	0.013	0.823	−0.005	0.927	0.003	0.955	0.003	0.951
Women (*n* = 326)										
Age	0.020	0.719	0.028	0.617	0.076	0.174	0.096	0.082	0.028	0.619
Body mass index	0.220	<.001	0.197	<.001	0.358	<.001	0.301	<.001	−0.011	0.840
Systolic blood pressure	0.125	0.024	0.111	0.045	0.076	0.170	0.069	0.213	0.029	0.596
Diastolic blood pressure	−0.039	0.484	−0.046	0.411	−0.063	0.259	−0.055	0.325	−0.008	0.880
Total cholesterol	−0.068	0.221	−0.039	0.482	−0.018	0.748	0.003	0.961	0.055	0.322
HDL cholesterol	−0.075	0.180	−0.050	0.375	−0.112	0.044	−0.087	0.121	0.000	0.994
Triglycerides	0.121	0.030	0.125	0.025	−0.009	0.877	0.030	0.594	0.077	0.167

*Abbreviations*: *HDL* high density lipoprotein, *FVC* forced vital capacity, *FEV1* forced expiratory volume in one second

The results of the multiple linear regression analyses of HDL cholesterol and pulmonary function are shown in Table 3. Among male adolescents, the level of HDL cholesterol showed significant negative associations with FVC (β = −0.010, *p* = 0.028), FEV1 (β = −0.010, *p* = 0.014), %FVC (β = −0.192, *p* = 0.036), and %FEV1 (β = −0.210, *p* = 0.017), even after adjusting for age, height, weight, smoking, alcohol drinking, physical activity, systolic blood pressure, total cholesterol, and triglycerides. Therefore, an increase of 1.0 mg/dL in HDL cholesterol was associated with a decrease of 10 mL in both FVC and FEV1. However, the same association was not significant in female adolescents. To evaluate whether socio-economic status has any effect on the association between HDL cholesterol and pulmonary function, we performed further analysis with additional adjustment for monthly household income level. However, the association did not change.

**Table 3:** Multiple linear regression between HDL cholesterol and pulmonary function

Variable	FVC, L		FEV1, L		Percent predicted FVC		Percent predicted FEV1	
	β	*p*	β	*p*	β	*p*	β	*p*
Males (*n* = 318)								
Model 1	−0.011	0.003	−0.009	0.005	−0.285	0.001	−0.247	0.001
Model 2	−0.008	0.039	−0.008	0.015	−0.172	0.024	−0.195	0.007
Model 3	−0.008	0.039	−0.008	0.014	−0.185	0.017	−0.209	0.004
Model 4	−0.010	0.028	−0.010	0.014	−0.192	0.036	−0.210	0.017
Model 5	−0.010	0.013	−0.010	0.013	−0.192	0.036	−0.209	0.017
Females (*n* = 326)								
Model 1	−0.003	0.312	−0.002	0.429	−0.088	0.141	−0.071	0.328
Model 2	−0.001	0.604	−0.001	0.787	−0.034	0.556	−0.011	0.873
Model 3	−0.002	0.535	−0.001	0.693	−0.037	0.519	−0.017	0.809
Model 4	0.002	0.497	0.002	0.464	−0.036	0.597	−0.015	0.858
Model 5	0.001	0.636	0.002	0.445	−0.046	0.499	−0.005	0.952

Model 1: unadjusted

Model 2: adjusted for age, height, and weight

Model 3: adjusted for age, height, weight, alcohol drinking, smoking, and exercise

Model 4: adjusted for age, height, weight, alcohol drinking, smoking, exercise, systolic blood pressure, total cholesterol, and triglycerides

Model 5: adjusted for age, height, weight, alcohol drinking, smoking, exercise, systolic blood pressure, total cholesterol, and triglycerides, and monthly household income

*Abbreviations*: *FVC* forced vital capacity, *FEV1* forced expiratory volume in one second

## Discussion

This study showed an association between HDL cholesterol and pulmonary function in males only. The findings remained consistent after adjusting for age, height, weight, physical activity, systolic blood pressure, and total cholesterol and triglyceride levels.

A recent study showed that elevated apolipoprotein M gene expression and higher levels of HDL cholesterol are associated with a lower FEV1/FVC ratio [12], corroborating our findings. The present study was not designed to determine the mechanisms leading to apolipoprotein M elevation, but it is known that the serum levels of apolipoprotein M and HDL cholesterol are positively correlated [17, 18]. A previous study showed that levels of HDL cholesterol were elevated in patients with chronic obstructive pulmonary disease compared with a reference participant group without disease [19]. In this regard, our results add to earlier findings that HDL cholesterol is also associated with impaired pulmonary function, independent of confounders, in healthy adolescents. However, in contrast to the current study, previous studies have suggested that serum levels of HDL cholesterol are positively correlated with pulmonary function in participants with and without asthma [20, 21]. In previous studies of the relationship between metabolic abnormalities and pulmonary function, elevated serum triglyceride and low HDL cholesterol were independent predictors of impaired pulmonary function [22, 23]. A cross-sectional study of adolescents found that those with low HDL cholesterol levels had lower FEV1/FVC ratios [24]. After adjusting for adiposity, HDL cholesterol remained a predictor of FEV1/FVC ratio.

Although the underlying mechanism is not yet understood, the composition of pulmonary surfactant might be a possible link between increased HDL cholesterol level and decreased pulmonary function. First, increasing HDL cholesterol could change the surface properties of pulmonary surfactant, a complex mixture of lipids and proteins. The mature lung contains approximately 300 million alveoli that each maintain their own function despite their very small radii [25]. This is because there is an air-liquid interface at which surface tension exists. Here, pulmonary surfactant reduces the surface tension in order to avoid collapse of alveolar architecture at the end of expiration and to assist the work of breathing [26, 27]. A recent study found that, of all serum components individually tested, HDL cholesterol modified the surface properties of surfactant [28]. Another possibility is that HDL cholesterol inhibits tumor necrosis factor-alpha (TNF-α) stimulated sphingosine kinase activity in endothelial cells, resulting not only in decreased sphingosine 1-phosphate production but also increased ceramide [12, 29]. Ceramide, the central molecule in the sphingolipid pathway, has been shown to induce apoptosis in experimental mouse models of emphysema [30]. In addition, a previous study has shown that increased levels of ceramide affect surfactant production [31]. Decreased pulmonary function could be due to HDL cholesterol-modified surface tension, as well as increased rigidity in the polar region of the surfactant.

In our study, the inverse association between HDL and pulmonary function was observed only in male adolescents. This sex-specific difference may be due to difference in absolute levels of HDL cholesterol. Women have higher HDL cholesterol levels compared with men of the same age. HDL cholesterol levels peak at 10–14 years of age in men and at 25–59 years of age in women [32]. A cohort study of 259 premenopausal women showed that both hormones and lipoprotein cholesterol levels varied across the menstrual cycle. Total and low-density lipoprotein cholesterol were highest during the follicular phase and declined during the luteal phase, whereas HDL cholesterol was highest at the time of ovulation [33]. It has been also reported that menopause is associated with lower FVC and FEV1 [34, 35]. When lipoprotein cholesterol levels vary across the menstrual cycle, it is difficult to observe the association between HDL and pulmonary function without considering the cyclic variations in lipoprotein levels in female adolescents. Consequently, sex hormones may act as a confounder influencing the observed sex differences in the association between HDL cholesterol and pulmonary function.

This study has some limitations. First, spirometry results depend not only on the true lung function of the participants but also on the quality of their test performance. Our analysis was limited to participants who had a minimum of three technically satisfactory pulmonary function measurements. Since interpretable spirometry data were collected irrespective of HDL cholesterol level, this should not have created significant bias. However, female adolescents were more likely to perform poorly on pulmonary function testing, which could underestimate their own lung function and attenuate the study results towards the null. Second, the cross-sectional design has some disadvantages compared with a cohort study, which is generally preferred to assess causal association. In this study, however, the underlying direction of causality is relatively distinct with regard to the effect of HDL cholesterol on pulmonary function. Third, most of the study participants were in the midst of puberty, thus hormonal status may influence the indices of anthropometry, as well as HDL cholesterol. Female adolescents enter puberty earlier and have a greater fat mass; these could be potential confounding factors when comparing the sexes. Finally, this study was conducted at a single high school and included one ethnic group; thus, the study findings cannot be generalized to other adolescent populations. However, the participants in this study were living in the same rural area, so there was no difference in environmental exposures that could affect lung function.

## Conclusions

This study suggests that high HDL cholesterol may be associated with decreased pulmonary function among healthy male adolescents. The observed sex differences in the association between HDL cholesterol and pulmonary function warrant further investigation.

## Additional file

Additional file 1: Study questionnaires used in the JSHS study. The supplementary file contains questionnaires about alcohol intake, smoking, physical activity, and socioeconomic status. (DOCX 269 kb)

## Abbreviations

FEV1: Forced expiratory volume in one second
FVC: Forced vital capacity
HDL: High density lipoprotein
LDL: Low density lipoprotein
