# Supplementary material for: Association between high-density lipoprotein cholesterol level and pulmonary function in healthy Korean adolescents: the JS high school study
Source: BMC Pulm Med. 2017 Dec 11;17:190. doi: 10.1186/s12890-017-0548-6 (PMC5725943; doi:10.1186/s12890-017-0548-6)
Supplement: Additional file 1: — Study questionnaires used in the JSHS study. The supplementary file contains questionnaires about alcohol intake, smoking, physical activity, and socioeconomic status. (DOCX 269 kb) [file 12890_2017_548_MOESM1_ESM.docx]

**Additional file 1. Study questionnaires used in the JSHS study**

| **Alcohol Intake** **Questions** consisted of simple questions about drinking in the present time and in the past:   1. Have you ever drunk alcohol at least once a month? 2. Yes 3. Does not take at all   The original Korean questionnaire  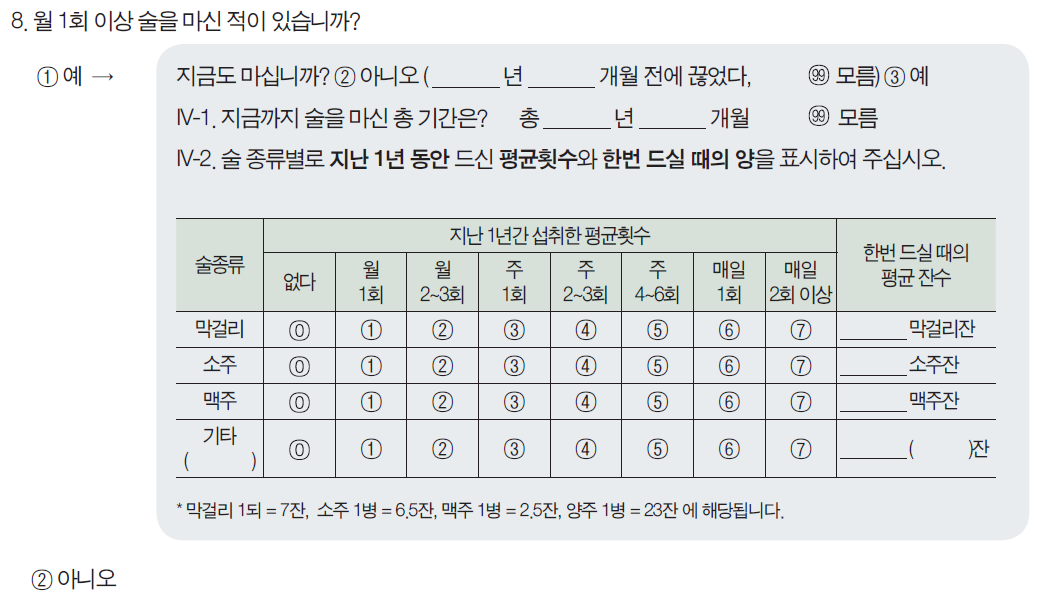 |
| --- |
| **Tobacco Smoking Questions** consisted of simple questions about smoking cigarettes in the present time and in the past:   1. Have you ever smoked cigarettes? (No means less than 5 packs of cigarettes or 100 cigarettes of tobacco in a lifetime) 2. How old were you when you first started regular cigarette smoking? 3. If you have stopped smoking cigarettes completely, how old were you when you stopped? 4. How many cigarettes do you smoke per day now?   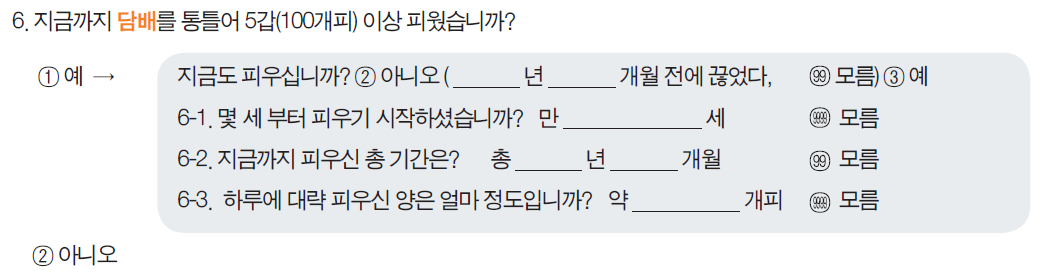The original Korean questionnaire |
| **Physical Activity Questionnaire** consisted of simple questions about regular exercise:   1. Do you exercise regularly at least once a week? 2. Yes 3. No   The original Korean questionnaire  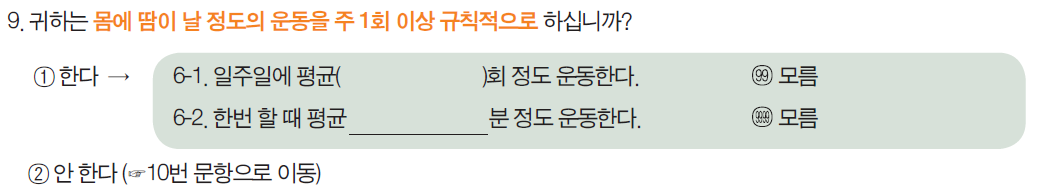 |
| **Monthly** **household** **income** consisted of simple questions about parents' income:   1. What is your average monthly income for your household? 2. Less than 1,000,000 Korean won 3. 1,000,000 – 1,990,000 Korean won 4. 2,000,000 – 2,990,000 Korean won 5. 3,000,000 – 3,990,000 Korean won 6. 4,000,000 – 4,990,000 Korean won 7. 5,000,000 – 5,990,000 Korean won 8. More than 6,000,000 Korean won   99) Do not wish to answer  The original Korean questionnaire  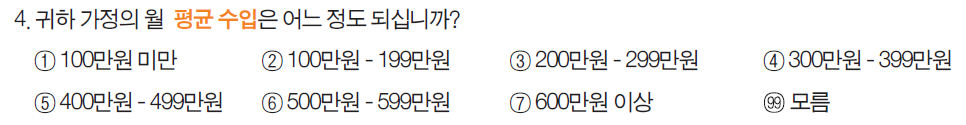 |
